# Exploratory study on the effectiveness of integrative neurocognitive remediation therapy (iNCRT) for cancer survivors

**DOI:** 10.1192/j.eurpsy.2022.1695

**Published:** 2022-09-01

**Authors:** A. Rogiers, D. Kyndt, S. Van Eycken, J.-C. Le Febvre, M. Brohee, C. Degols, C. Fontaine, B. Neyns, C. Kornreich

**Affiliations:** 1 CHU Brugmann, Psychiatry, Brussels, Belgium; 2 UZ Brussels, Oncology, Brussels, Belgium

**Keywords:** cancersurvivor, cognitive remediation therapy, cognitive impairment, Neurocognitive function

## Abstract

**Introduction:**

Cancer survivors frequently report suffering from neurocognitive impairment, that persists after physical recovery from their disease. Cognitive impairment is associated with important emotional disturbances, socio-professional consequences and diminished quality of life.

**Objectives:**

This observational study aims to assess the effectives of an integrative neurocognitive remediation therapy (iNRCT), offered as a 12-week program (1day/week), organized within our Cognitive Remediation Clinic. The iNCRT combines personalized computerized cognitive training and neurocognitive strategy training, with group sessions of physical exercise, mindfulness, and cognitive behavior therapy (CBT).

**Methods:**

The assessment before and after NCRT includes neuropsychological testing (10 subtests), assessment of daily functioning and subjective neurocognitive function (NCF).

**Results:**

Out of 16 eligible cancer survivors, 12 patients were recruited and 11 completed the iNCRT; median age 53 years [range, 41-71]; 3 patients had a prior history of a central nervous system tumor, 5 patients of breast cancer, 2 patients of stage-IV melanoma, and 1 patient of gastric cancer. After iNCRT subjective NCF did not improve significantly (p=0.13) according to the Cognitive Failure Questionnaire. However neuropsychological assessment revealed an improvement on ≥ 1 impaired subtest in all patients; 6 patients improved on ≥ 4 impaired subtests. Improvement was most prominent in long-term verbal and visual memory, working memory and executive function. All patients reported a clinical benefit in their daily function after completion of iNCRT.

**Conclusions:**

Our iNRCT, which combines personalized neurocognitive training with physical exercise, mindfulness and CBT can be an effective therapeutic model for treating neurocognitive impairment in cancer survivors, with a clinically relevant impact on their daily function.

**Disclosure:**

No significant relationships.

